# Secondary school students’ use and perceptions of textbooks in mathematics learning: A large-scale investigation in China

**DOI:** 10.3389/fpsyg.2023.1132184

**Published:** 2023-03-06

**Authors:** Tianzhuo Jiang, Shuwen Li

**Affiliations:** School of Mathematics and Statistics, Northeast Normal University, Changchun, China

**Keywords:** textbook use, mathematics textbooks, mathematics learning, curriculum resources, secondary school students, Chinese mathematics education

## Abstract

Students’ use of textbooks is the key link of students engaged and learned curriculum and has received much attention recently. However, existing studies were mainly case studies or small-scale investigations and few addressed the context of China. Hence, this study provided a general overview of mathematics textbook use by Chinese secondary students through a large-scale investigation. Using a mixed-method approach, we collected the quantitative data from 2,145 students in eight provinces through a questionnaire survey and the qualitative data from 20 students and 8 teachers by the interviews. The results revealed that (1) Chinese students relied heavily on mathematics textbooks and pointedly used a portion of components in textbooks, mainly kernels, examples, and exercises; (2) Chinese students used mathematics textbooks for various but typical reasons, particularly to understand basic knowledge and skills, and showed self-regulation and teacher-mediation behind their use; and (3) Chinese students had a positive view about textbook use in mathematics learning, especially in developing mathematical knowledge, skills, and abilities. Furthermore, there were significant differences in mathematics textbook use among different students in terms of school regions, grade levels, and teachers’ demographic variables. Finally, explanations and implications of the results were discussed.

## 1. Introduction

As the main vehicles of curriculum content and the key resources for teaching and learning, textbooks have always been a hot topic in educational research. In recent decades, various issues related to mathematics textbooks have been researched and discussed, including their composition, use, and history (Schubring and Fan, 2018). Among the issues, research on mathematics textbook use has received increasing attention and become an important part of topic study groups of the 13th (Fan et al., 2018) and 14th International Congress on Mathematical Education and one of the hot topics of the 2nd (Schubring et al., 2018), 3rd (Rezat et al., 2019) and 4th International Conference on Mathematics Textbook Research and Development.

As educational researchers have increasingly recognized the important role that textbooks play in mathematics teaching and learning, how mathematics textbooks are incorporated into teaching and learning has been explored. With regard to teachers’ use of mathematics textbooks, numerous studies have been conducted from various perspectives, including but not limited to offering a framework for characterizing and studying teachers’ interactions with curriculum materials (Remillard, 2005), examining how teachers used their curriculum resources to teach new mathematics standards (Polly, 2017), and so on (Nicol and Crespo, 2006; Grave and Pepin, 2015; Olsher and Cooper, 2021). On the contrary, studies on students’ use of mathematics textbooks have not been paid adequate attention, with the results being largely discrete and non-inclusive (Wang and Fan, 2021, p. 2). Meanwhile, it is clear that most of previous studies were carried out in the broad international context but in a small scale (Fan et al., 2013; Rezat, 2013) and little research focused Chinese context. Moreover, researchers have discussed some factors that influence students’ textbook use, but there is a lack of comprehensive research regarding the demographic and contextual factors influencing students’ use of mathematics textbooks.

In this study, we attempt to provide an overview of Chinese secondary students’ use and perceptions of mathematics textbooks and its factors through a large-scale investigation. We believe that focusing on Chinese students’ use of mathematics textbooks can make a distinctive and important contribution to research on students’ use of textbooks. First, China is the most populous developing country with a large number of students in junior high schools (Ministry of Education of the People’s Republic of China, 2022b) in the world. Second, the tradition of Chinese culture, especially Confucian culture, has played an important role in modern mathematics education. Third, the impacts of textbook use on students’ mathematics learning might, to some extent, offer an explanation for the well-known fact that Chinese students have outperformed their western counterparts in the international comparative studies of mathematics achievements such as the Trends in International Mathematics and Science Study and the Program for International Student Assessment (Lin et al., 2018). Thus, this study proposed the following questions:

(1) What is the current status of Chinese students’ use of mathematics textbooks?
(2) Are there differences among Chinese students in terms of school regions, student genders, grade levels, and teachers’ demographic variables in mathematics textbook use?

## 2. Literature review

### 2.1. Students’ use of mathematics textbooks

Students’ use of mathematics textbooks is common, but the possible relationship between students and mathematics textbooks is complex and dynamic. According to existing research, text-reading theory, reader-oriented theory, and activity theory have generally been used to analyze students’ use of mathematics textbooks. Based on text-reading theory, the “use” is understood as reading, a transaction between mathematics textbooks (written curriculum) and students (Berger, 2019). In reader-oriented theory, the student is viewed as actively constructing meaning from mathematics textbooks through the reading process, which is shaped and constrained by the intentions of the author, the beliefs of the reader, and the qualities the text requires the reader to possess (Weinberg and Wiesner, 2011). From the perspective of activity system, students’ textbook use refers to the activities that are primarily associated with textbooks in students’ mathematics learning, such as reading and practicing (Rezat and Sträßer, 2013; Wang and Fan, 2021). To capture current situation of Chinese students’ use of mathematics textbooks, this study employed the definition of students’ textbook use based on activity theory. Activity theory is a descriptive psychological theory that studies and explains the emergence and development of human psychology with activity as its logical starting point and central category. Activity theory has its roots in the classical philosophy of Kant and Hegel, the contemporary philosophy of Marx and Engels, and the cultural-historial psychology of Vygotsky, Leontev, and Luria (Jonassen and Rohrer-Murphy, 1999). As a powerful psychological framework rather than a methodology, the six elements in activity theory: subject, artifact, object, community, rules, and division of labor directed this study. Specifically, we focused on how students interact with mathematics textbooks in social contexts by situating students, textbooks, and mathematics into the subject-artifacts-object triangle (Vygotsky, 1978). Meanwhile, we considered the influence of the region (social contexts), students’ gender and grade level (subject), and teachers’ variables (community, rules, and division of labor) on students’ use of mathematics textbooks.

As mentioned earlier, several studies have been conducted from different facets to investigate how students used mathematics textbooks. Fan et al. (2004) focused on the following aspects of students’ textbook use, including (1) the frequency and timing of textbook use, (2) how students used different textual parts, (3) to what extent students thought that textbook use was important for learning mathematics, and (4) whether and why students changed their ways of textbook use compared to the last semester. They found that textbooks were students’ main learning resources for both in-class exercises and homework. Besides, Rezat’s series of studies dealt with students’ use of mathematics textbooks at school level. Rezat (2011) presented five self-regulated learning activities that involved textbooks in learning mathematics: (1) task and problem solving, (2) practice, (3) acquisition of new knowledge, (4) interest-driven activities, and (5) meta-cognitive learning activities. Meanwhile, he (2013) also conducted a study on how 74 students in two German secondary schools used their mathematics textbooks for practice. In Weinberg et al.’s 2012 study, students in introductory mathematics courses were surveyed to answer which parts of the texts they used, and when and why they used textbooks. Moreover, Thomas (2013) examined how students in two classrooms taught by the same teacher used print and digital formats of the Algebra 1 textbook. The results indicated that most students used a small portion of the resources and features in the textbook, tended to view the textbook primarily as a source for homework, and rarely bothered to develop examples and texts in class. From a comparative perspective, Wang and Fan (2021) proposed seven indicators to investigate the use of mathematics textbooks by students in Shanghai and England, including the frequency, duration, timing, purpose, and motivation of textbook use, access to textbooks, and the influence of textbook use on mathematics learning.

In summary, most studies focused on how students used mathematics textbooks in English-speaking countries and few addressed the context of China. Moreover, existing studies were mainly case studies or small-scale investigations. Meanwhile, various indicators related to the word “use” were proposed to embody students’ use of mathematics textbooks. Against this background, this study aims to conduct a large-scale investigation of Chinese secondary students’ use and perceptions of mathematics textbooks. According to Jonassen and Rohrer-Murphy’s (1999) process for applying activity theory, we focused on when and why activities occur, what are used to perform activities, and what are the outcomes of activities. And based on previous studies, we established a conceptual framework (Table 1) consisted of four subdimensions with seven indicators to capture how Chinese students use mathematics textbooks.

**TABLE 1:** Subdimensions and indicators of current situation of students’ textbook use.

Subdimensions	Indicators
Length of time of textbook use	The days of textbook use in a week
	The duration of textbook use in a day
Frequency of textbook use	The frequency of textbook use at different timing
	The frequency of using different components
Reasons of textbook use	The purposes of textbook use
	The motivation of textbook use
Perceptions of textbook use	The recognition of the impacts of textbook use on students’ mathematics learning

The first four indicators are proposed to understand to what extent students rely on mathematics textbooks in their learning. Two of the indicators are the days of textbook use in a week and the duration of textbook use in a day. Meanwhile, the frequency of textbook use at different timing refers to the frequency of students’ textbook use before, in, and after class and before the examination. The frequency of using different components refers to the frequency of using “introductions,” “exploratory tasks,” “kernels (definitions, theorems, and formulas),” “worked examples,” “tips,” “exercises and problems,” “summaries,” “mathematics activities,” and “reading materials” which were divided based on textbook editors’ suggestions.

Furthermore, this study defines the purpose of textbook use as specific learning activities related to mathematics textbooks, which include “preview,” “revision,” “doing homework,” “doing extra exercises,” “in-class learning and exercises,” “looking up examples, answers, and references,” and “looking up definitions, theorems, and formulas” (Weinberg et al., 2012; Rezat, 2013; Wang and Fan, 2021). Moreover, the indicator “motivation” is proposed to understand the reasons behind textbook use, which was drawn upon Amabile et al.’s (1994) scale, Ryan and Deci’s (2000) definition and structure, and Wang and Fan’s (2021) construct. The motivation consists of intrinsic motivation (enjoyment and challenge) and extrinsic motivation, which is composed of two aspects: external regulation (teacher-mediation and parent-supervision) and self-regulation (students’ recognition of the impacts of textbook use on “grades,” “knowledge, skills, and abilities,” and “thinking methods, activity experience, and emotions and values”).

In addition, students’ perceptions of textbook use are adopted to understand to what extent students recognize the impacts of textbook use on mathematics learning. Previous research has explored the effects of curriculum resources as instruments on students’ achievement (Van den Ham and Heinze, 2018; Sievert et al., 2021), conceptual understanding (Rezat, 2021), beliefs (Moyer et al., 2018; Kersey, 2019), identities (Macintyre and Hamilton, 2010), and levels of participation (Ewing, 2006). Also based on the aims of Mathematics Curriculum Standards for Compulsory Education (Ministry of Education of the People’s Republic of China, 2011, 2022a), this study reflects the influence of textbook use on mathematics learning in whether textbook use helps students improve mathematics grades, master mathematics knowledge, enhance mathematical skills, develop mathematical abilities, understand mathematical thinking methods, gain mathematical activity experience, and shape mathematical emotions and values.

### 2.2. Factors on students’ use of mathematics textbooks

Students’ use of mathematics textbooks is a learning activity which is influenced by both internal and external factors of activity. Internal factors of activity consists of student factors and textbook factors. For student factors, Fan et al. (2004) found the majority of students (78%) have changed their ways of textbook use from the first year to the second year in junior high schools. One of the main reasons for the change was that students realized mathematics more important for them. Students’ grade levels and beliefs of mathematics become important factors which influence students’ use of mathematics textbooks. Regarding textbook factors, students’ use of mathematics textbooks is influenced by content, structure and formats of textbooks (Jukić Matić and Glasnović Gracin, 2016). Österholm (2006) recruited 61 secondary and 34 university students to compare their reading comprehension of one historical text and two mathematical texts, both of which presented basic concepts of group theory, but one did it using mathematical symbols, whereas the other only used natural language. The vertical comparison of the students’ prior knowledge with their results in the reading test revealed a similarity in reading comprehension between the mathematical text without symbols and the historical text as well as a difference between the two mathematical texts. Similarly, Rezat’s (2013) study showed how textbook users were influenced by the way mathematics was presented in the textbooks. Besides, Thomas (2013) identified there were differences between digital and print textbook use by students in two classrooms taught by the same teacher.

Among external factors of activity, country or region factors and teacher factors are the important factors behind students’ use of mathematics textbooks. In their comparative study, Wang and Fan (2021) found that there were significant differences between Shanghai and England regarding the role that textbooks played as curriculum resources in students’ mathematics learning. Meanwhile, Fan et al. (2004) found there were significant differences in students’ use of mathematics textbooks, especially the frequency of using different components, between two regions in China. Furthermore, teachers’ mediated intervention plays an important role in students’ use of mathematics textbooks (Griesel and Postel, 1983; Rezat, 2006, 2009). Although many instructors might not clearly tell their students how to use the textbook, students reported that they used it more productively when they believed they had been asked to do so (Weinberg et al., 2012). Teachers as the mediators decided which textbooks to use; when and where the textbook was to be used; which sections of the textbook to use; the sequencing of topics in the textbook; the ways in which students engaged with the text; the level and type of teacher intervention between students and textbooks; and so on (Pepin and Haggarty, 2001). Rezat (2012) summarized the conceptualization of six different ways teachers mediate textbook use in matrix and stated that all three dimensions are intertwined in a concrete mediation of textbook use.

Summarily, students’ use of mathematics textbooks is affected by various factors, including students’ grade levels, text formats, school regions, and teachers. However, little research has taken the influence of teachers’ demographic variables (teachers’ gender, education level, title, teaching experience, and experience in teaching with textbooks) on students’ use of mathematics textbooks into consideration. Hence, this study intends to present a systematic investigation of the factors that influence Chinese secondary students’ textbook use in learning mathematics.

## 3. Materials and methods

### 3.1. Instruments

In this study, mixed methods were used to collect data on students’ textbook use in learning mathematics through a questionnaire survey and the interviews with students and teachers.

#### 3.1.1. Questionnaire

The questionnaire, designed on the basis of the conceptual framework, consisted of five parts with 27 questions. The first part related to students’ demographic information, including region, gender and grade level. The second part was about the days of textbook use in a week and the duration of textbook use in a day. The options for two questions were intervals. The third part contained 13 questions, four of which referred to the frequency of textbook use before, in, and after class and before the examination and another nine of which referred to the frequency of using “introductions,” “exploratory tasks,” “kernels,” “worked examples,” “exercises and problems,” “tips,” “summaries,” “mathematics activities,” and “reading materials.” A five-point Likert scale was used to capture students’ options (e.g., never, seldom, sometimes, often, always). In the fourth part, two questions focused on the reasons for textbook use: one related to specific activities students engaged in when learning mathematics with textbooks and the other involved possible motivations behind textbook use. Students were asked to select all options that applied to their purposes and motivation of textbook use. Finally, the remaining seven questions addressed the impacts of textbook use on improving mathematics grades, mastering mathematics knowledge, improving mathematical skills, developing mathematical abilities, understanding mathematical thinking methods, gaining mathematical activity experience, and shaping mathematical emotions and values. A five-point Likert scale was employed to gather students’ recognition of the impacts of textbook use on their mathematics learning (e.g., not helpful, not very helpful, slightly helpful, helpful, very helpful). After the questionnaire was drafted, a panel of mathematics education researchers was invited to review it and they were highly positive about the instrument, indicating reasonable validity. Meanwhile, the reliability test of the questionnaire determined using Cronbach’s alpha yielded a value of 0.950, indicating high reliability.

#### 3.1.2. Interview outline

Furthermore, we conducted interviews with students and teachers to complement the questionnaire data and gather more in-depth details about how students use mathematics textbooks. The outline of student interview was in line with the four subdimensions of the questionnaire. The first part included two questions: (1) How frequently and when do you use textbooks in your mathematics learning? What other curriculum resources do you use to learn mathematics? (2) Do you think that textbook use is helpful in learning mathematics? And why? The second part consisted of two different situations: in class and out of class (mainly at home), but with the same three questions: (1) Do you use the textbook at the request of teachers/parents or on your own initiative? (2) Which parts of the textbook do you use? (3) For what purposes do you use the text components? According to Jukić Matić and Glasnović Gracin (2016) questions and Rezat’s (2012) conceptualization, the protocol of teacher interview was developed with three questions: (1) How do you usually prepare a mathematics lesson? (2) What proportion of your teaching content in class comes from mathematics textbooks? And why are the contents in textbooks added, deleted and adjusted? (3) Whether you ask students to use textbooks inside and outside mathematics classrooms? Which parts and why are students asked to use?

### 3.2. Data collection and analysis

#### 3.2.1. Data collection

After obtaining ethical approval to conduct the research, students who participated in the questionnaire were selected through multi-stage sampling. In the first stage, according to stratified sampling, three provinces were separately selected from eleven provinces in the east and twelve provinces in the west, and two provinces were selected from eight provinces in the midlands. Secondly, one city from each sampled province was selected by cluster sampling. In the third phase, one school was randomly selected in each city. Finally, at least one class of each grade level was selected in sampled schools. At the same time, we also collected the basic information of mathematics teachers who taught the sampled students. We distributed 2,300 questionnaires to all participating students and obtained 2,145 valid questionnaires, which was a response rate of 93.3%. Table 2 shows the profiles of the participating students.

**TABLE 2:** The profiles of students surveyed.

Variables		Categories	N	Percentage
Region		East	728	33.94%
		Middle	653	30.44%
		West	764	35.62%
Gender		Male	1106	51.56%
		Female	1039	48.44%
Grade level		7th grade	747	34.83%
		8th grade	902	42.05%
		9th grade	496	23.12%
Students taught by different teacher groups	Gender	Male	285	13.29%
		Female	1860	86.71%
	Education level	Bachelor’s degrees or lower	1468	68.44%
		Master’s degrees or higher	677	31.56%
	Title	Primary	1169	54.50%
		Middle	390	18.18%
		Senior	586	27.32%
	Teaching experience	≤5 years	1230	57.34%
		6–15 years	143	6.67%
		>15 years	772	35.99%
	Experience in textbook teaching	≤5 years	1230	57.34%
		>5 years	915	42.66%

For an in-depth understanding of the quantitative data, we randomly selected 20 students not involved in the questionnaire to conduct focus group interviews online and selected 8 their mathematics teachers by purposive sampling to conduct individual interviews on line or by phone calls. The interviewed students and teachers covered different comparison groups to ensure representativeness. Specifically, Table 3 shows the basic information of teachers interviewed and among the 20 students, 8 were males and 12 were females; 7 were from the east, 7 were from the midlands, and 6 were from the west; 8 were from the 7th grade, 9 were from the 8th grade, and 3 were from the 9th grade. During the whole process of survey and interviews, we keep the data strictly confidential and anonymous to fully protect the privacy of all participating students and teachers.

**TABLE 3:** Basic information of teachers interviewed.

	Gender	Level of education	Title	Experience of teaching	Experience of teaching with textbooks
T1	Male	Bachelor	Primary	≤5 years	≤5 years
T2	Female	Master	Primary	≤5 years	≤5 years
T3	Female	Bachelor	Primary	≤5 years	≤5 years
T4	Female	Master	Middle	6–15 years	>5 years
T5	Male	Master	Middle	6–15 years	>5 years
T6	Male	Master	Senior	>15 years	>5 years
T7	Male	Bachelor	Senior	>15 years	>5 years
T8	Female	Bachelor	Senior	>15 years	>5 years

#### 3.2.2. Data analysis

The questionnaire data were analyzed by descriptive and inferential statistics, and presented numerically and graphically. We first calculated the percentage or mean and standard deviation for each item in the questionnaire. To explore factors influencing students’ use of mathematics textbooks, we distinguished several comparison groups of students in terms of school regions, student genders, grade levels, and teachers’ demographic variables. Then, we conducted *T*-tests, One-way analysis of variance (ANOVA), or Chi-square tests on each item to examine whether there were statistically significant differences in textbook use among students of different groups.

For the qualitative data from the interviews, we first coded the interviewed students as S1 to S20 and interviewed teachers as T1 to T8 to protect their privacy. Then, two researchers conducted independent transformation and interpretation of interview transcripts by identifying different indicators of textbook use to obtain more information about students’ textbook use.

## 4. Results

### 4.1. Current situation of students’ textbook use

#### 4.1.1. Students’ length of time of mathematics textbook use

**FIGURE 1:**
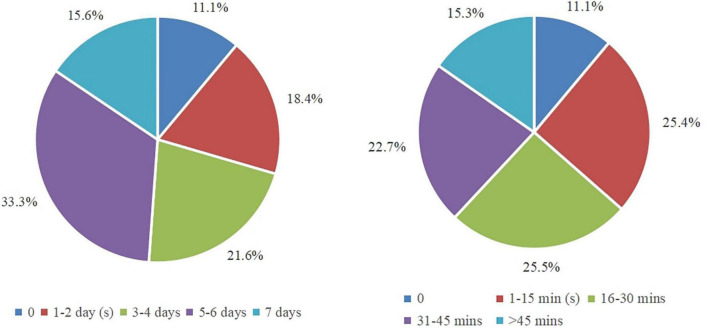
Percentage distributions of the days and duration of textbook use.

Figure 1 shows the days students used mathematics textbooks in a week and the duration students used mathematics textbooks in a day. Nearly half (48.9%) of students used textbooks at least 5 days per week, 40% used textbooks 1–4 days per week, and a minority (11.1%) didn’t use textbooks. Meanwhile, the majority (63.5%) of students used textbooks more than 15 min per day and 25.4% used textbooks within 15 min per day. During the interviews, 14 students reported that they used textbooks more than 5 days per week and another 6 students used textbooks 1–4 days per week. Meanwhile, 3 students reported that they used textbooks more than 30 min per day, 9 students used textbooks 16–30 min per day, and the remaining used textbooks less than 15 min. The results revealed that Chinese students used mathematics textbooks for many days per week and longtime per day, indicating that they relied heavily on mathematics textbooks.

#### 4.1.2. Students’ frequency of mathematics textbook use

**FIGURE 2:**
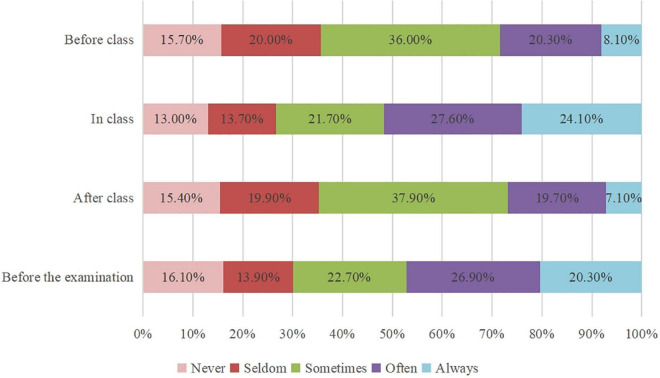
Percentage distributions of the frequency of textbook use at different timing.

As shown in Figure 2, about 15% of students never used mathematics textbooks before, in, and after class and before the examination. And about half of students at least often used textbooks in class (51.7%) and before the examination (47.2%), while more than half of students seldom or sometimes used textbooks before (56.0%) and after (57.8%) class. In the interviews, 12 students reported that they at least often used textbooks in class. Meanwhile, 16 students stated that they sometimes or often used textbooks before and after class, which appeared more frequent use than the questionnaire data. In contrast to the data surveyed, only seven students reported that they sometimes or often used textbooks before the examination. Merely one student (S4) said: “I always used textbooks before the final and midterm examinations and weekly tests.” The results revealed that Chinese students used mathematics textbooks more frequently in class and before the examination than before and after class.

**FIGURE 3:**
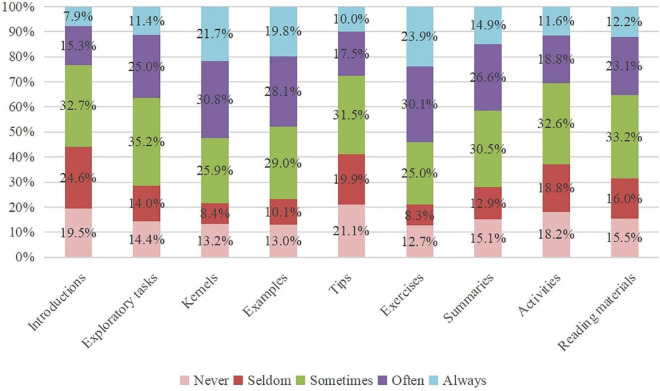
Percentage distributions of the frequency of using different components.

According to Figure 3, about 10% of students always used introductions, exploratory tasks, tips, mathematics activities, and reading materials, and 15–20% of students never used these parts. However, about half of students at least often used kernels, worked examples, exercises and problems, and summaries, and about 15% of students never used these parts. During the interviews, all students stated that they at least sometimes used kernels, worked examples, and exercises and problems. Meanwhile, exploratory tasks, tips, summaries, and reading materials were rarely mentioned (less than 5 students, respectively), and introduction and mathematics activities were not mentioned at all. The results revealed that Chinese students used core content in mathematics textbooks more frequently than content with guidance and auxiliary.

#### 4.1.3. Students’ reasons of mathematics textbook use

**FIGURE 4:**
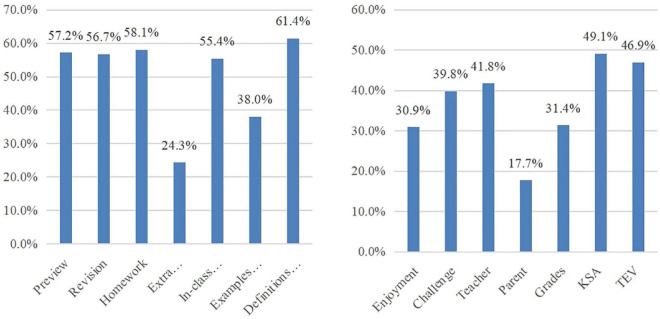
Percentage distributions of the purposes and motivation of textbook use.

As shown in Figure 4, mathematics textbooks were typically used by more than half of Chinese students to preview, revise, do homework, learn and exercise in class, and look up definitions, theorems, and formulas. Consistent with the data surveyed, all interviewees mentioned the above five purposes of textbook use. Besides, 11 interviewed students used textbooks to look up examples and 3 interviewed students used textbooks to do extra exercises. The four typical motivations behind textbook use in turn were their perceptions of textbook’s helpfulness for “knowledge, skills, and abilities” and “thinking methods, activity experience, and emotions and values,” teacher-mediation, and challenges. In the interviews, all students pointed out that textbook use is helpful for their mathematics learning and their teachers asked them to use textbooks in class, but no one mentioned that they would feel fulfilled when they solved difficult problems in textbooks. Besides, six students reported that their parents asked them to use textbooks. For example, S18 said: “My mother told me ‘I was too busy to tutor you, so you should use textbooks more because there were all you have ever studied in textbooks.”’ The results revealed that Chinese students used mathematics textbooks for various but typical purposes and motivation.

#### 4.1.4. Students’ perceptions of mathematics textbook use

Table 4 shows the means, standard deviation, and rank of the influence of textbook use on mathematics learning. The mean scores of seven items ranged from 3.502 to 3.992, which were greater than 3.500, implying positive impacts of textbook use on mathematics learning. Textbook use is perceived to be most helpful for mathematics knowledge, followed by mathematical skills, abilities, thinking methods, activity experience, values and emotions, and grades. During the interviews, all students stated that textbook use is helpful for developing mathematical knowledge, skills, and abilities. Besides, 4 students said that “When I met the problems that I could not solve, I would read the examples and exercises to find the methods.” One student (S3) emphasized that “I would lose marks because of incomplete steps, so I would often read and imitate the steps of the examples in mathematics textbooks, which could help me reduce the loss of marks.” While, no one explicitly mentioned that textbook use is helpful for gaining activity experience and shaping emotions and values. The results revealed that Chinese students had a positive view about textbook use in mathematics learning, especially in developing basic knowledge, skills, and abilities.

**TABLE 4:** The M, SD, and rank of the influences of textbook use on mathematics learning.

	Grades	Knowledge	Skills	Abilities	Methods	Experience	Emotions
M	3.502	3.992	3.595	3.570	3.546	3.539	3.509
SD	1.0112	0.9622	1.0364	1.0780	1.0424	1.0525	1.0940
Rank	7	1	2	3	4	5	6

### 4.2. Analysis of differences across student groups

To examine the effects of different factors on students’ use of mathematics textbooks (see Table 5), Chi-square tests were conducted for the length of time and reasons of textbook use among different comparison groups. In frequency and perceptions of textbook use, *T*-tests were conducted across students’ gender, teachers’ gender, education level and experience in teaching with textbooks and ANOVA tests were conducted across school region, grade level, and teachers’ title and teaching experience. Meanwhile, the interviews with students and teachers were conducted to offer explanations for the differences in students’ textbook use across different comparison groups.

**TABLE 5:** Significant differences among different students in mathematics textbook use.

			Length of time of textbook use (*X*^2^)		Frequency of textbook use at different timing (*F*/*t*)					
			**Days/week**	**Min/day**	**Before class**		**In class**	**After class**	**Before the exam**	
Region			178.494***	213.551***	65.383***		138.744***	49.633***	63.890***	
Gender				11.790*						
Grade level			304.491***	295.354***	65.049***		76.174***	71.828***	109.397***	
Students taught by different teacher groups		Gender	101.287***	101.023***	-6.579***		-7.487***	-7.850***	-8.996***	
		Education level		11.790*			5.669***		2.732**	
		Title	83.835***	84.073***	20.520***		38.101***	19.673***	27.334***	
		Teaching experience	44.292***	44.916***	5.738**		12.303***	6.513**	16.571***	
		Experience in textbook teaching	27.594***	26.827***	-2.284*		-4.189***	-2.961**	-5.063***	
		**Frequency of using different components (*F*/*t*)**								
		**Introductions**	**Exploratory tasks**	**Kernels**	**Worked examples**	**Tips**	**Exercises**	**Summaries**	**Mathematics activities**	**Reading materials**
Region		93.453***	71.440***	44.199***	48.498***	86.122***	47.318***	77.717***	88.473***	90.636***
Gender		2.461*								
Grade level		66.435***	99.824***	105.036***	101.806***	41.867***	124.357***	76.431***	68.733***	84.459***
Students taught by different teacher groups	Gender	-7.262***	-8.023***	-7.785***	-5.903***	-5.590***	-7.558***	-6.625***	-7.665***	-7.310***
	Education level	3.687***	2.727**	2.172*		2.856**		3.556***	2.924**	3.767***
	Title	25.964***	27.778***	22.129***	23.697***	21.712***	26.103***	32.293***	31.667***	39.953***
	Teaching experience	12.813***	16.137***	14.465***	11.926***	7.754***	13.528***	11.754***	15.970***	17.035***
	Experience in textbook teaching	-4.540***	-4.381***	-4.033***	-2.630**	-2.597**	-3.476**	-4.275***	-4.390***	-4.989***
		**Reasons of textbook use (*X*^2^)**		**Perceptions of textbook use (*F*/*t*)**						
		**Purpose**	**Motivation**	**Grades**	**Knowledge**	**Skills**	**Abilities**	**Methods**	**Experience**	**Emotions**
Region		60.026***	83.796***	133.537***	34.292***	102.836***	98.243***	90.797***	88.878***	104.515***
Gender			30.467***							
Grade level		38.400***	44.766***	39.815***	16.753***	27.523***	37.529***	37.167***	37.880***	33.920***
Students taught by different teacher groups	Gender			-5.380***	-4.553***	-4.107***	-4.056***	-4.767***	-5.521***	-5.026***
	Education level	17.117**		5.538***	3.224**	4.169***	4.183***	3.976***	2.358*	3.922***
	Title		26.564**	34.482***	12.660***	36.632***	33.792***	35.138***	26.102***	29.821***
	Teaching experience			11.284***	5.911**	8.962***	10.056***	13.555***	7.969***	6.723**
	Experience in textbook teaching		14.587*	-4.161***	-2.055*	-2.711**	-3.667***	-4.080***	-2.136*	-2.582*

*p < 0.05, **p < 0.01, and ***p < 0.001.

#### 4.2.1. School region: Access to and types of other curriculum resources

The test results showed that there were statistically significant differences in the length of time, frequency, reasons, and perceptions of textbook use among the three regions. Specifically, students from the midlands used textbooks significantly more time, more frequently, and more for typical reasons and thought significantly more highly of textbooks in mathematics learning than those from the west, whereas students from the west were significantly greater than those from the east in four dimensions of textbook use. During the interviews, we found that the region differences in textbook use were related to access to and types of other curriculum resources. According to students’ responses, other curriculum resources could be classified into information and communication technologies (ICTs), supplementary educational books, and school-based learning materials and their deciders could be the publisher, local teaching and research section, school, teacher, parent, or student (see Table 6). The interview data revealed that students from the east had more access to other more curriculum resources, so that they used textbooks less time, less frequently, and less for typical reasons and thought less highly of textbooks in mathematics learning than those from the west, whereas students from the west were less than those from the midlands in four dimensions of textbook use.

**TABLE 6:** The classifications of other curriculum resources and their deciders.

	Students from the east	Students from the midlands	Students from the west
ICTs	Smartphone (parent/student), computer (parent/student), website (student), PowerPoint (teacher), Bilibili (student), Geometer’s Sketchpad (student)	Smartphone (parent/student), website (student), PowerPoint (teacher)	Smartphone (parent/student), website (student), PowerPoint (teacher), Bilibili (student)
Supplementary educational books (SEB)	Workbook (publisher), SEB1 (school), SEB2 (student), SEB3 (student)	Workbook (publisher), SEB4 (school)	Workbook (publisher), SEB5 (school), SEB6 (school)
School-based learning materials	Class notes (teacher), guiding case (teacher), Workbook (local teaching and research section)	Class notes (teacher)	Class notes (teacher), guiding case (teacher)

#### 4.2.2. Student genders

The test results showed that there were no statistically significant differences in the length of time and frequency of textbook use between the two genders, except that boys used textbooks significantly more minutes per day than girls and used introductions in mathematics textbooks significantly more frequently than girls. Also, there were no statistically significant differences in reasons and perceptions of textbook use between the two genders, except that it is significantly more enjoyable for boys to use textbooks than girls. Similarly, we have not found that there were evident differences in textbook use between the two genders in the interviews.

#### 4.2.3. Grade level: Curriculum content and students’ mathematics knowledge base

The test results showed that there were statistically significant differences in the length of time, frequency, reasons, and perceptions of textbook use among the three grade levels. Specifically, the seventh graders used textbooks significantly more time, more frequently, and more for typical reasons and thought significantly more highly of textbooks in mathematics learning than the eighth graders, whereas the eighth graders were significantly greater than the ninth graders in four dimensions of textbook use. According to the interviews, we thought the grade differences in textbook use were due to curriculum content and students’ mathematics knowledge base.

For example, S9 said: “The content in the seventh grade is relatively simple and some have been learned in primary school. When in the eighth grade, the amount and difficulty of curriculum content are grater. So I often used the textbook to look up definitions, theorems, and formulas.” Also, S1 and S14, the eighth graders, expressed the same views. Meanwhile, S10 said: “The content in the ninth grade is more compositive. When learned new content or solved the problems, I need more prior knowledge than before. So I often used the textbook to review.”

S5, S6, and S8 said: “When learned parallel lines, congruent triangles, and parallelogram, I used the textbook more frequently to look up examples. Because I had to imitate the steps of proof to do homework and further understand reasoning.”

S15 and S17 said: “Compared to the last semester, I think I have understood and mastered the content after learning in class and doing homework (not in the textbook) after class. So I hardly used the textbook and occasionally used the textbook only when I cannot deal with the problems well.”

The results of the above interviews revealed that breadth and difficulty of curriculum content, specific content areas, and students’ mathematics knowledge base were the factors that affected students’ textbook use. Specifically, as the grade changed, students used textbooks more time, more frequently, and more for typical reasons and thought more highly of textbooks in mathematics learning with curriculum content broader and more difficult, but students used textbooks less time, less frequently, and less for typical reasons and thought less highly of textbooks in mathematics learning with the foundation of mathematics knowledge more solid. Moreover, when it came to figures and geometry, students used textbooks more frequently to understand methods and imitate process of proof. Overall, the phenomenon of “students’ use of mathematics textbooks declined with higher grade level” was prominent.

#### 4.2.4. Teachers’ demographic variables: Teachers’ textbook use and interventions with students

The test results showed that there were statistically significant differences in the length of time and frequency of textbook use among students taught by teachers with different demographic variables, except the days of textbook use in a week, the frequency of textbook use before and after class, and the frequency of using examples and exercises in textbooks between students taught by teachers with different levels of education. Regarding the reasons of textbook use, there were no statistically significant differences among students taught by teachers with different demographic variables, except the purposes of textbook use between students taught by teachers with different levels of education and the motivation of textbook use among students taught by teachers with different titles and with different experiences in teaching with textbooks. And for the perceptions of textbook use, there were statistically significant differences among students taught by teachers with different demographic variables.

Specifically, students taught by female teachers used textbooks significantly more time, more frequently, and more for typical reasons and thought significantly more highly of textbooks in mathematics learning than students taught by male teachers. Similarly, students taught by teachers with bachelor’s degrees or lower used textbooks significantly more time, more frequently, and more for typical reasons and thought significantly more highly of textbooks in mathematics learning than students taught by teachers with master’s degrees or higher. In contrast, students taught by teachers with middle titles or with 6–15 years of experience in teaching mathematics used textbooks significantly more time, more frequently, and more for typical reasons and thought significantly more highly of textbooks in mathematics learning than students taught by teachers with primary and senior titles or with less than 5 years and more than 15 years of experience in teaching mathematics. Meanwhile, students taught by teachers with more than 5 years of experience in teaching with textbooks used textbooks significantly more time, more frequently, and more for typical reasons and thought significantly more highly of textbooks in mathematics learning than students taught by teachers with less than 5 years of experience in teaching with textbooks. In the interviews, we found these results were related to teachers’ ways of textbook use and ways of intervention with students. According to teachers’ responses, teachers’ ways of textbook use involved direct use, indirect use, and absence of use (Jukić Matić and Glasnović Gracin, 2016) and teachers’ ways of intervention with students were intertwined in direct (specific/general)/indirect and obligatory/voluntary (Rezat, 2012), as shown in Table 7.

**TABLE 7:** Teachers’ ways of textbook use and ways of interventions with students.

	Teachers’ ways of textbook use			Teachers’ ways of intervention with students
	**Direct use**	**Indirect use**	**Absence of use**	
T1	80%	15%	5%	Direct, specific, obligatory, and voluntary
T2	75%	15%	10%	Indirect and voluntary
T3	85%	15%		Direct, specific, obligatory
T4	70%	25%	5%	Direct, specific and general, obligatory
T5	65%	25%	10%	Indirect and voluntary
T6	65%	30%	5%	Direct, general, obligatory, and voluntary
T7	70%	25%	5%	Direct, general, obligatory, and voluntary
T8	75%	20%	5%	Direct, specific, obligatory, and voluntary

From the results of interviews, female teachers and teachers with bachelor’s degrees or lower were more likely to use mathematics textbooks directly and mediate students directly and specifically. For example, T3 said: “The examples, exercises, and homework were directly from textbooks… and I always asked students to read the kernels in class.” Thus, their students used textbooks more frequently and thought more highly of textbooks in mathematics learning. However, teachers with middle titles, teachers with 6–15 years of experience in teaching mathematics, and teachers with more than 5 years of experience in teaching with textbooks were more likely to use mathematics textbooks less directly and more indirectly and mediate students more generally and indirectly. For example, T4 said: “I would adjust the amount and sequence of exploratory tasks in textbooks when I think they are not suitable for students… and I would also advise students to use textbooks when need help for tasks.” But their students still used textbooks more frequently and thought more highly of textbooks in mathematics learning.

## 5. Discussion

### 5.1. Mathematics textbooks: Traditional but important curriculum resources for students’ mathematics learning

Due to rapid technological, cultural, and economic development in China over the past 20 years, ICTS, supplementary educational books and school-based learning materials have become increasingly available in Chinese classrooms and families. As traditional curriculum resources, mathematics textbooks are still important tools for Chinese secondary students to learn mathematics which is consistent with previous studies (Fan et al., 2004; Wang and Fan, 2021). Specifically, Chinese students relied heavily on mathematics textbooks. Quite a few students used mathematics textbooks both in school and at home and not only on weekdays but also on weekends. Meanwhile, they used mathematics textbooks at different timing, especially in class and before the examination. And they pointedly used a portion of components in textbooks, mainly kernels, examples, and exercises. This finding is in line that of with existing studies (Weinberg et al., 2012; Thomas, 2013). Besides, Chinese students used mathematics textbooks for various but typical reasons. They used mathematics textbooks particularly to preview, revise, do homework, learn and exercise in class, and look up definitions, theorems, and formulas, which was largely affected by the Confucian tradition of ‘learning the new by repeating the old’ and the two-basic teaching of basic knowledge and basic skills. And they showed self-regulation (Wang and Fan, 2021) and teacher-mediation behind their use. Furthermore, Chinese students had a positive view about textbook use in mathematics learning, especially in developing mathematical knowledge, skills, and abilities. But, they were less positive about the impact of textbook use on grades, mainly because they had more access to other curriculum resources and ignored that using textbooks could improve their grades by developing their basic knowledge and basic skills.

### 5.2. Differences in students’ use of mathematics textbooks across demographic factors

The results revealed that there existed significant differences in students’ use of mathematics textbooks in terms of school regions, grade levels, and teachers’ demographic variables, except student genders. Regarding school regions, students from the east relied significantly less on textbooks, used textbooks significantly less for typical reasons, and had significantly less positive views about textbook use in mathematics learning than students from the west, who were less than students from the midlands. This finding is different from Fan et al.’s (2004) conclusions that students from Fuzhou in the east used textbooks and their components more frequently than students from Kunming in the west. We think this contradiction is partly related to access to and types of other curriculum resources, behind that is local economic and educational development level. In fact, China is divided into the east, midlands, and west according to economic development level of every province ranging from high to low and geographical location. In the early 20th century, students from two regions had less access to other curriculum resources, which was not the main factor affecting students’ use of mathematics textbooks at that time. Whereas after 20 years of economic and educational rapid development, students from three regions have more access to other various curriculum resources, which has a huge impact on textbook use. Further studies should be conducted to explore how economic development affects students’ textbook use.

With regard to student genders and grade levels, there were fewer significant differences between gender groups, which revealed that student genders might not be the main factor affecting students’ textbook use. In terms of grade levels, the higher graders relied significantly less on textbooks, used textbooks significantly less for typical reasons, and had significantly less positive views about textbook use in mathematics learning than the lower graders. From the interviews, we found this result was related to breadth and difficulty of curriculum content, specific content areas, and students’ mathematics knowledge base, which was both consistent with and complementary to the existing study (Fan et al., 2004). Fan et al. (2004) found the reasons why students have changed their use of mathematics textbooks compared to the previous semester were mainly breadth and difficulty of curriculum content, students’ beliefs of mathematics, editors of textbooks, teachers, and parents. Curriculum content broader and more difficult with the grade level increasing is understandable because curriculum content in Chinese mathematics textbooks are organized in spiral sequence form. Meanwhile, there are more reasoning and proof in figures and geometry than numbers and algebra and statistics and probability (Ministry of Education of the People’s Republic of China, 2011) and examples in textbooks provide students scientific ideas, authoritative process, and standard steps of the proof to imitate and understand reasoning, which were the reasons why students used textbooks more frequently and had more positive views about textbook use when they learned figures and geometry. In addition, students’ developmental levels represented by students’ mathematics knowledge are also the factors that influence students’ textbook use. As grade levels changed, students with more solid foundation of mathematics knowledge and more mature beliefs could more effectively use textbooks to integrate new knowledge with prior experience, so that they used textbooks less frequently and had less positive views about textbook use. Under the influence of student factors and content factors, students’ use of mathematics textbooks declined with higher grade level. Whether student factors play a more important role in students’ textbook use than content or textbook factors needs to be further examined.

Regarding teachers’ demographic variables, students taught by female teachers, by teachers with bachelor’s degrees, by teachers with middle titles, by teachers with 6–15 years of experience in teaching mathematics, or by teachers with more than 5 years of experience in teaching with textbooks relied significantly more on textbooks and had significantly more positive views about textbook use in mathematics learning than students taught by male teachers, by teachers with master’s degrees, by teachers with primary and senior titles, by teachers with 0–5 and more than 15 years of experience in teaching mathematics, or by teachers with 0–5 year of experience in teaching with textbooks, respectively. But there were fewer differences in reasons of textbook use among students taught by teachers with different demographic variables. This finding was related to teachers’ ways of textbook use and ways of intervention with students, behind that were teachers’ knowledge and beliefs about mathematics textbooks (Rezat, 2013; Jukić Matić and Glasnović Gracin, 2016). According to the interviews, female teachers and teachers with bachelor’s degrees or lower, who were more likely to regard textbooks as the decisive tools to complete the teaching task, would tend to use mathematics textbooks faithfully and explicitly ask students to use specific section. Although teachers with more than 5 years of experience in teaching with textbooks have more indirect use of textbooks and general mediation on students, experienced textbook users who had wealthy knowledge and mature beliefs about textbooks would urge students to read the main body in textbooks more (Fan et al., 2004). Therefore, these explained why their students used textbooks more frequently and thought more highly of textbooks in mathematics learning. But compared to novice and experienced teachers, knowledge and beliefs about mathematics textbooks of teachers with middle titles and teachers with 61–5 years of experience in teaching mathematics are in the stage of development and formation. They were more likely to use mathematics textbooks less directly and more indirectly and mediate students more generally and indirectly. Why their students used textbooks more frequently and thought more highly of textbooks in mathematics learning could not be well explained in this study. This might be due to the small number of interviewed teachers with middle titles and with 6–15 years of experience in teaching mathematics and no classroom observations. Further research should be conducted to explore what cause such a difference.

### 5.3. Implications

Although this study focused on Chinese context, some results and discussions may have general implications. From the perspective of the subject and artifact in activity system, mathematics textbooks should meet students’ individual development and diverse needs. On the one hand, students have more access to more various curriculum resources. On the other hand, different groups of students use mathematics textbooks in different ways. These mean that mathematics textbooks will be easily at a disadvantage in students’ resource system. Therefore, it is a key issue to monitor the quality of curriculum resources (Wang and Fan, 2021), further to make mathematics textbooks play a more important role in the resource system from students’ different learning needs.

From the perspective of the community in activity system, students should be taught to use mathematics textbooks autonomously and creatively to improve learning quality through teachers’ mediation. Teachers also proved to be important in the students’ use of mathematics textbooks. Teachers’ knowledge and beliefs about textbooks strongly shape their practice and decide when, where, and which sections of the textbook were to be used by students. Thus, it is necessary for teachers to help students realize the importance and significance of mathematics textbooks and textbook use. It can promote students to know long-term and short-term goals for textbook use and develop the awareness and ability to regulate their textbook use.

### 5.4. Limitations and future directions

Finally, we should point out that the data collected were mainly based on self-report and no classroom observations were employed due to the unexpected impact of the COVID-19 pandemic, which are the limitations of this study. According to this study, Chinese students use a variety of curriculum resources to learn mathematics and teachers’ knowledge and beliefs about textbooks behind teachers’ demographic variables are the potential factors that affect students’ textbook use. Hence, future research should explore how students in different economic, social, and cultural contexts incorporate various curriculum resources in mathematics learning. In fact, many researchers expressed similar concerns about this issue. For example, Glasnović Gracin and Jukić Matić (2021) have explored teachers’ and students’ use of textbooks and other resources during the process of educational reform in Croatia and Fan et al. (2022) have studied Shanghai students’ access, use, and perceptions of ICTs in learning mathematics. Moreover, examining the relationship between teachers’ knowledge and beliefs about textbooks and students’ textbook use could be a direction for further studies.

## Data availability statement

The raw data supporting the conclusions of this article will be made available by the authors, without undue reservation.

## Ethics statement

The studies involving human participants were reviewed and approved by the Northeast Normal University Academic Ethics Committee. The participants provided their written informed consent to participate in this study.

## Author contributions

TJ contributed to the literature review and theoretical background, collected and analyzed the data, and wrote the original draft of the manuscript. SL contributed to the construction of concepts and index framework, questionnaire preparation, introduction, and methodology, and revised the manuscript. Both authors contributed to the article and approved the submitted version.
